# The Role of Epac in Cancer Progression

**DOI:** 10.3390/ijms21186489

**Published:** 2020-09-05

**Authors:** Nadine Wehbe, Hasan Slika, Joelle Mesmar, Suzanne A. Nasser, Gianfranco Pintus, Serine Baydoun, Adnan Badran, Firas Kobeissy, Ali H. Eid, Elias Baydoun

**Affiliations:** 1 Department of Biology, American University of Beirut, P.O. Box 11-0236 Beirut, Lebanon; nww04@mail.aub.edu (N.W.); jm104@aub.edu.lb (J.M.); 2 Department of Pharmacology and Therapeutics, Faculty of Medicine, American University of Beirut, P.O. Box 11-0236 Beirut, Lebanon; hgs09@mail.aub.edu; 3 Department of Pharmacology, Beirut Arab University, P.O. Box 11-5020 Beirut, Lebanon; san413@bau.edu.lb; 4 Department of Biomedical Sciences, University of Sharjah, P.O. Box 27272 Sharjah, UAE; gpintus@sharjah.ac.ae; 5 Department of Biomedical Sciences, University of Sassari, Viale San Pietro 43, 07100 Sassari, Italy; 6 Department of Radiology, American University of Beirut, P.O. Box 11-0236 Beirut, Lebanon; sb63@aub.edu.lb; 7 Department of Basic Sciences, University of Petra, P.O. Box 961343, Amman 11196, Jordan; abadran@uop.edu.jo; 8 Department of Biochemistry and Molecular Genetics, Faculty of Medicine, American University of Beirut, P.O. Box 11-0236, Beirut, Lebanon; firasko@gmail.com; 9 Department of Pharmacology and Therapeutics, Faculty of Medicine, American University of Beirut, P.O. Box 11-0236, Beirut, Lebanon

**Keywords:** Epac, cancer, reactive oxygen species (ROS), cAMP, PKA

## Abstract

Cancer continues to be a prime contributor to global mortality. Despite tremendous research efforts and major advances in cancer therapy, much remains to be learned about the underlying molecular mechanisms of this debilitating disease. A better understanding of the key signaling events driving the malignant phenotype of cancer cells may help identify new pharmaco-targets. Cyclic adenosine 3′,5′-monophosphate (cAMP) modulates a plethora of biological processes, including those that are characteristic of malignant cells. Over the years, most cAMP-mediated actions were attributed to the activity of its effector protein kinase A (PKA). However, studies have revealed an important role for the exchange protein activated by cAMP (Epac) as another effector mediating the actions of cAMP. In cancer, Epac appears to have a dual role in regulating cellular processes that are essential for carcinogenesis. In addition, the development of Epac modulators offered new routes to further explore the role of this cAMP effector and its downstream pathways in cancer. In this review, the potentials of Epac as an attractive target in the fight against cancer are depicted. Additionally, the role of Epac in cancer progression, namely its effect on cancer cell proliferation, migration/metastasis, and apoptosis, with the possible interaction of reactive oxygen species (ROS) in these phenomena, is discussed with emphasis on the underlying mechanisms and pathways.

## 1. Introduction

Cancer is one of the leading causes of morbidity and mortality around the world. In 2018, it attributed to approximately 9.6 million deaths worldwide, and this number is expected to increase by 71.5% in 2040 [1,2]. According to the International Agency for Research on Cancer (IARC), the most common causes of cancer deaths globally are cancers of lung, colorectum, stomach, liver, and breast [3]. Although there are no specific causes of cancer, 30–50% of cancers can be prevented by avoiding or amending risk factors such as tobacco use, alcohol consumption, unhealthy diet, and low physical activity [4]. Early diagnosis and adequate treatment increase the chance of curing many cancers. A number of cancer treatments involves a combination of surgery, chemotherapy, and radiation therapy. These treatments, however, are not always successful, mainly due to the development of chemoresistance and relapse [4]. Hence, there is an urge to seek and develop alternative therapeutic approaches for the treatment of cancer. 

Cyclic adenosine 3′,5′-monophosphate (cAMP) plays a crucial role in mediating intracellular signaling transduction in response to external or internal stimuli. It regulates several cellular processes, including cell proliferation, migration, differentiation, and apoptosis as well as many processes involved in physiology and pathophysiology [5,6,7,8,9,10,11,12,13,14,15]. cAMP is synthesized by the action of adenylyl cyclase (AC) isoforms, either membrane-bound or soluble, using ATP as a precursor [16]. The intracellular level of cAMP is regulated by the activity of ACs, phosphodiesterases (PDEs), and A-kinase anchoring proteins (AKAPs). PDEs degrade cAMP into 5′-AMP, thus lowering the level of cAMP inside the cell and terminating its signal transduction [17]. AKAPs, on the other hand, are scaffolding proteins that ensure the compartmentalization of cAMP and its signaling components by sequestering them into subcellular domains [18]. This compartmentalization is important to achieve specific and efficient activation of the second messenger, cAMP, in response to a stimulus [19,20]. 

cAMP classically mediates its actions via the activation of protein kinase A (PKA) [21]. A new downstream effector was later identified by two independent research groups in 1998 [22,23]. This effector is currently known as exchange protein activated by cAMP (Epac), which acts as a guanine nucleotide exchange factor (GEF) for the Ras family members, Rap1 and Rap2. There are two isoforms of Epac proteins, designated as Epac1 and Epac2, which differ in their expression profile. While Epac1 is ubiquitously expressed with particular abundancy in the heart and the kidneys [22], Epac2 is prominently expressed in the brain and the adrenal glands [22,23]. Alternative splicing of Epac2 gene gives rise to three variants with differences in structure and tissue-specific expression. Epac2A is expressed in the brain, the pancreas, and the pituitary [23,24], Epac2B is expressed in the adrenal glands [25], and Epac2C is liver specific [26]. 

In addition to Rap1/2, several studies have identified other downstream effectors of Epac proteins. For instance, Epac1 directly interacts with R-Ras, another member of the Ras superfamily of small GTPases, which in turn stimulates the activation of phospholipase D (PLD) [27]. Epac also directly activates c-Jun N-terminal Kinase (JNK) [28], Rim-2 [29], Rim-2-related protein Piccolo [30], and SUR1 [31] in a Rap-independent manner. Rim-2, a Rab3-interacting protein, plays a role in docking vesicles to the plasma membrane, and the Epac2-Rim-2-SUR1 complex was found to be involved in exocytosis machinery [32]. Moreover, Epac binds to the light chain 2 (LC2) of the microtubules associated protein (MAP1A), which acts as an adaptor protein to facilitate the interaction of Epac with the cytoskeleton and also enhances Epac-mediated activation of Rap1 [33,34]. A study has shown that Rit small GTPase functions downstream of cAMP/Epac in a Rap1-independent manner; however, neither Epac 1 nor Epac2 directly activates Rit [35]. This suggests that there is another signal transduction pathway that mediates the activation of Rit via Epac proteins. Although studies have revealed the interaction of Epac proteins with a number of effector proteins, little is known about these effectors. Thus, future in silico modeling studies are warranted to further identify potential signaling molecules downstream of Epac.

**Figure 1:**
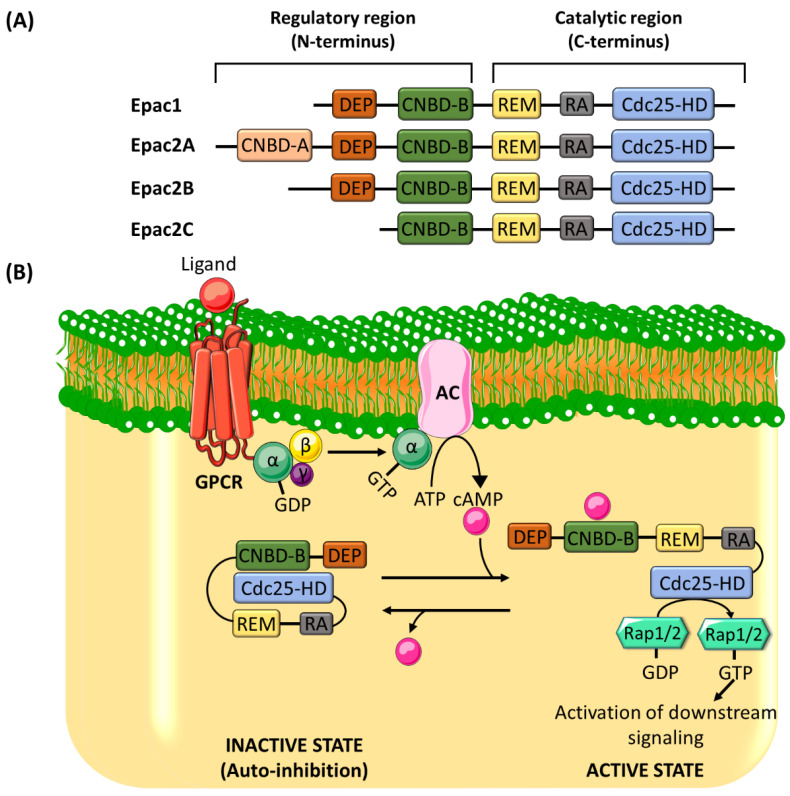
(**A**) The structure of exchange protein activated by cyclic adenosine 3′,5′-monophosphate (cAMP) (Epac) proteins. Epac is made up of a catalytic region, which comprises three domains, and a regulatory region, which consists of two domains. The domains of the catalytic region are Ras-exchange motif (REM), Ras-association (RA), and cell division cycle 25 homology domain (Cdc25-HD), and the domains of the regulatory region are dishevelled/Egl-10/pleckstrin (DEP) and cAMP-nucleotide binding-B domain (CNBD-B). There are structural differences in the regulatory regions between Epac1 and Epac2. Epac2A has two cAMP binding domains, CNBD-A and CNBD-B. The DEF domain is missing in Epac2C. (**B**) The mechanism of Epac proteins activation. After its activation by the Gα subunit of Gs protein, AC produces cAMP from ATP. The binding of cAMP to the CNBD-B within the regulatory region of Epac induces a conformational change, which is required to alleviate the autoinhibitory effect. Rap1/2 is subsequently allowed to bind to the catalytic domain (Cdc25-HD), where it is activated by the guanine nucleotide exchange factor (GEF) activity of Epac. AC: adenylyl cyclase; GPCR: G-protein coupled receptor.

Epac proteins are multi-domain polypeptides made up of a C-terminal catalytic region and an N-terminal regulatory region (Figure 1A). The catalytic region consists of a Ras-exchange motif (REM) domain, a Ras-association (RA) domain, and a cell division cycle 25 homology domain (Cdc25-HD) [36]. While Cdc25-HD is responsible for the GEF activity of Epac [37], REM and RA domains play roles in stabilizing the active conformation of Epac and targeting Epac to the membrane, respectively [38,39]. The regulatory region is made up of a cAMP-nucleotide binding-B domain (CNBD-B) and a dishevelled/Egl-10/pleckstrin (DEP) domain [36]. As its name implies, the CNBD-B acts as the binding site for cAMP, whereas the DEP domain plays a role in translocating Epac from the cytosol to the plasma membrane [40]. Epac adopts an autoinhibitory conformation, where the interaction between the regulatory CNBD and the catalytic Cdc25-HD locks Epac in an inactive state and hinders the accessibility of Rap to the catalytic domain [22]. The binding of cAMP to CNBD induces a conformational change, releasing the autoinhibition and exposing Cdc25-HD for the Rap [40] (Figure 1B).

Although the discovery of Epac is relatively new, studies have revealed its involvement in regulating myriad cellular processes such as cell proliferation, apoptosis, migration, and adhesion in various body systems [36,41]. Epac has been shown to mediate its action via modulating key signaling pathways involved in cell mitogenesis, cytoskeletal remodeling, inflammation, and oxidative stress. In general, Epac and PKA may act independently, antagonistically, or synergistically to regulate cellular processes. For instance, cAMP-induced stabilization of endothelial cell–cell junctions is mediated by both Epac and PKA through the activation of two parallel and independent pathways. While PKA requires integrin-mediated cell adhesion to promote endothelial integrity, Epac/Rap1 signaling does not [42]. On the other hand, Epac and PKA can act synergistically to regulate cell proliferation in thyroid cells [43] and vascular smooth muscle cells [44,45].

While the role of cAMP/PKA signaling pathway is well established in cancer progression [46,47], the association of Epac with cancer is still emerging. Studies have demonstrated a dual role of the novel cAMP effector, Epac, in cancer, where it could promote or attenuate cancer initiation and progression. This makes Epac a promising target for therapeutic approaches in treating cancer. Due to its controversial role in cancer progression, the aim of our review is to analyze and critically discuss the effects of Epac on cancer cell proliferation, migration/metastasis, and apoptosis by focusing on the underlying molecular mechanisms and targeted pathways.

## 2. Role of Epac in Cancer Cell Proliferation

Epac has a dual role in regulating cancer growth and progression. While the majority of studies describe a pro-proliferative role of Epac in many cancers, others depict a protective role (Table 1). The discrepancy in these findings could be due to differences in cell types or even differences in the genome and the transcriptome among cell lines of the same cancer. Epac regulates cancer cells proliferation and survival in a wide variety of cancers by targeting key signaling pathways involved in cell mitogenesis, inflammation, and metabolic reprogramming.

**Figure 2:**
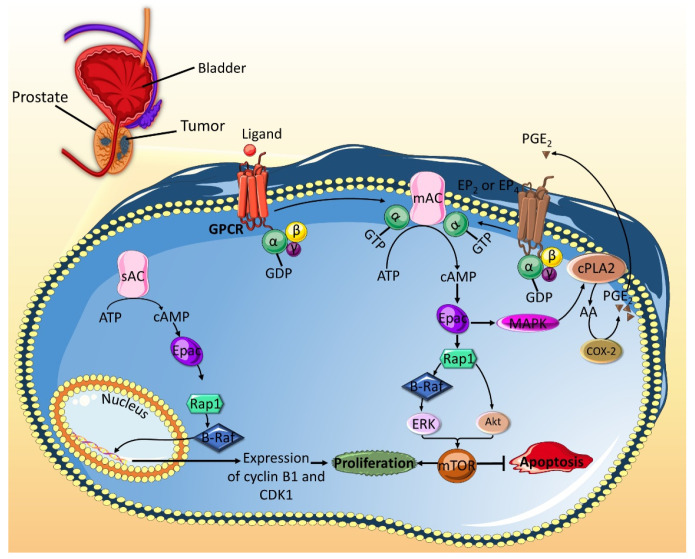
Epac promotes cell proliferation and attenuates apoptosis in prostate cancer. The activation of the soluble type 10 adenylyl cyclase (sAC) leads to the generation of cAMP, which in its turn activates Epac. Epac then induces the Rap1-dependent activation of B-Raf, which subsequently leads to increased expression of cell cycle proteins cyclin B1 and CDK1. These proteins mediate the G2/M phase transition of the cell cycle and promote proliferation. On the other hand, activation of the mAC leads to cAMP generation and subsequent activation of Epac. Epac can then activate the B-Raf/ERK and the Akt pathways, which both converge on mTOR. The activated mTOR mediates pro-proliferative and anti-apoptotic roles. These effects are amplified by the pro-inflammatory role that Epac can play. Indeed, Epac activates MAPK, which the activates cPLA2. The latter changes membrane phospholipids into AA, which is acted upon by COX-2 to produce PGE2. The PGE2 produced by prostate cancer cell can diffuse to the tumor microenvironment to activate EP2 and EP4 receptors on the cell itself or neighboring cells. EP2 and EP4 receptors are associated with G stimulatory proteins, which lead to further activation of mAC and subsequent accumulation of cAMP/Epac. AA: arachidonic acid; COX-2: cyclooxygenase-2; cPLA2: cytosolic phospholipase A2; EP2: PGE2 receptor 2; EP4: PGE2 receptor 4; mAC: membrane bound adenylyl cyclase; PGE2: prostaglandin E2.

Numerous studies evidently show that Epac enhances prostate cancer cells proliferation (Figure 2). Epac activates the extracellular response kinase 1/2 (ERK1/2) and the phosphoinositide 3-kinase (PI3K)/protein kinase B (Akt) signaling pathways, both of which converge at the mammalian target of rapamycin (mTOR) signaling [62,63]. mTOR is a serine/threonine kinase that plays a key role in enhancing cell proliferation [67]. Epac could also promote mTOR-mediated increase in cell proliferation by acting as a pro-inflammatory modulator, which augments the expression of chronic inflammation markers, such as cytosolic phospholipase A2 (c-PLA2) and cyclooxygenase-2 (COX-2), and production prostaglandin E2 (PGE2). A possible explanation could be that, in Epac-stimulated cells, arachidonic acid, which is generated by increased activity of c-PLA2, gets converted into PGE2 by COX-2. Binding of PGE2 to its receptors, PGE2 receptor 2 (EP2) and 4 (EP4) subtypes, activates cAMP/Epac/Rap1 pathway, which promotes the activation of mTOR signaling [61]. Another mechanism by which Epac stimulates prostate cancer cells proliferation is by Rap1-mediated increase in the level of cell cycle regulators, such as cyclin B1 and CDK1, which play a role in the transition of cells from G2 to M phase and promote mitogenesis [64].

While most reports suggest that Epac promotes cancer cells proliferation in prostate cancer, one study showed that Epac inhibits cell proliferation in PC-3 and DU 145 prostate cancer cell lines by inhibiting MAPK pathway and therefore decreasing DNA synthesis [60]. It was later suggested that this contradictory finding could be due to the activation of PKA by the Epac-specific agonist, 8-(4-chlorophenylthio)-2′-O-methyl cyclic AMP (8-pCPT), also known as 007. Although 8-pCPT’s affinity for Epac is 100-fold greater compared to PKA, the inhibitory effects mediated by 8-pCPT were rescued by PKA inhibitors, H89 and PKI, but were not affected by Epac silencing [68].

**Figure 3:**
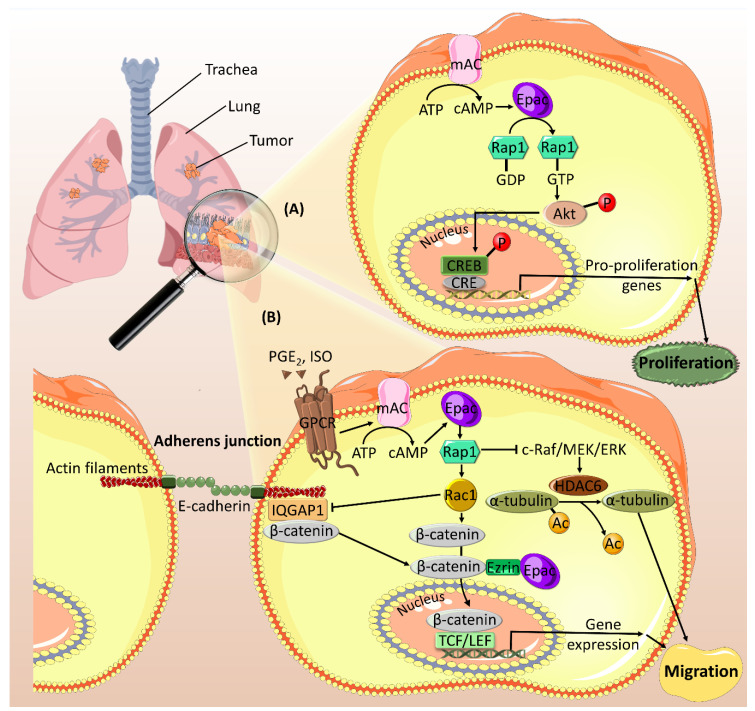
Epac promotes cell proliferation (**A**) and migration (**B**) in lung cancer. (**A**) Epac activates its downstream effector, Rap1, which increases the phosphorylation of Akt. Activated Akt mediates the phosphorylation of CREB in the nucleus. Upon its phosphorylation, CREB binds to CRE site on the DNA promoting the transcription of genes involved in cell proliferation. (**B**) Epac mediates PGE2- and ISO-induced cell migration by regulating different downstream effectors. PGE2 mediates cAMP-induced activation of Epac via its receptor EP2. Epac activates Rap1 and Rac1, which binds to its effector, IQGAP1, leading to its dissociation from the adherens junction. Subsequently, β-catenin is also dissociated and translocated to the perinuclear regions where it interacts with Epac via the adaptor protein Ezrin. This interaction mediates β-catenin entrance into the nucleus. In the nucleus, β-catenin binds to TCF/LEF transcription factors and activates the transcription of pro-migratory genes. On the other hand, ISO-mediated Epac/Rap1 activation inhibits c-Raf/MEK/ERK signaling, leading to an increase in the expression of HDAC6. HDAC6 deacetylates α-tubulin and promotes cell migration. Ac: acetyl group; CRE: cAMP response element; CREB: cAMP response element-binding protein; IQGAP1: IQ-motif containing GTPase activating protein 1; ISO: isoproterenol; PGE2: prostaglandin E2; TCF/LEF: T cell factor/lymphoid enhancer factor.

Moreover, Epac has a positive role in promoting lung cancer cells proliferation (Figure 3A). Recently, a pro-mitogenic role of Epac has been revealed in in vivo and in vitro models of lung cancer via the activation of Rap1 and Akt signaling [55]. In this context, Epac, but not PKA, has been found to mediate its effect by activating the cAMP response element-binding protein (CREB), which plays a role in enhancing cell proliferation and reducing apoptosis [69]. Furthermore, Epac has been shown to mediate cAMP-induced inhibition of DNA damage repair in lung cancer by promoting the degradation of X-ray repair cross-complementing protein 1 (XRCC1) [56]. XRCC1 is involved in DNA damage repair pathway, which has pro- and anti-carcinogenic roles depending on the stages of cancer development and progression [70]. Therefore, the effect of Epac-mediated inhibition of XRCC1 should be further investigated to confirm the role of Epac in lung cancer.

A recent study showed that Epac could act synergistically with PDE4, an enzyme associated with the incidence of multiple tumors [71], in promoting rectal carcinoma [65]. Although the exact mechanism was not identified, the researchers found correlations between PDE4, Epac, cyclin E1, and the gap junction protein, connexin-43 (Cx43), in tissues obtained from patients diagnosed with invasive rectal carcinoma [65]. Cyclin E1 plays a role in cell cycle G1/S phase transition, and its overexpression usually promotes tumorigenesis. Cx43, on the other hand, has a dual role in cancer progression. Although reports revealed a suppressing role of Cx43 in cancer, others reported an increase in its expression and membrane localization in metastatic lesions of multiple cancer types [72]. Nonetheless, one could speculate that Epac and PDE4 increase the expression of cyclin E1 and Cx43, leading to uncontrolled cell growth and metastasis in rectal carcinoma. Furthermore, Epac promotes cell proliferation in ovarian cancer, both in vitro and in vivo, by activating PI3K/Akt/Cyclin D1/CDK4 pathway [58]. Epac has been shown to enhance cell growth and survival in other cancer types, such as pancreatic [59], gastric [54], and breast cancer [53], yet the underlying mechanisms remain to be elucidated.

PDE4 is also expressed in brain tumors and promotes their growth [73]. Contrary to its role in rectal carcinoma, Epac/Rap1 along with PKA mediate cell cycle arrest induced by the PDE4 inhibitor, rolipram, in glioblastoma [51]. Additionally, another study demonstrated that Epac promotes glioblastoma regression by mediating the inhibition of MAPK activity [52]. Similarly, Epac exhibits an anti-proliferative role in clear renal cell carcinoma in mediating vasoactive intestinal peptide (VIP)-induced inhibition of cell proliferation through the PI3K pathway [66].

**Figure 4:**
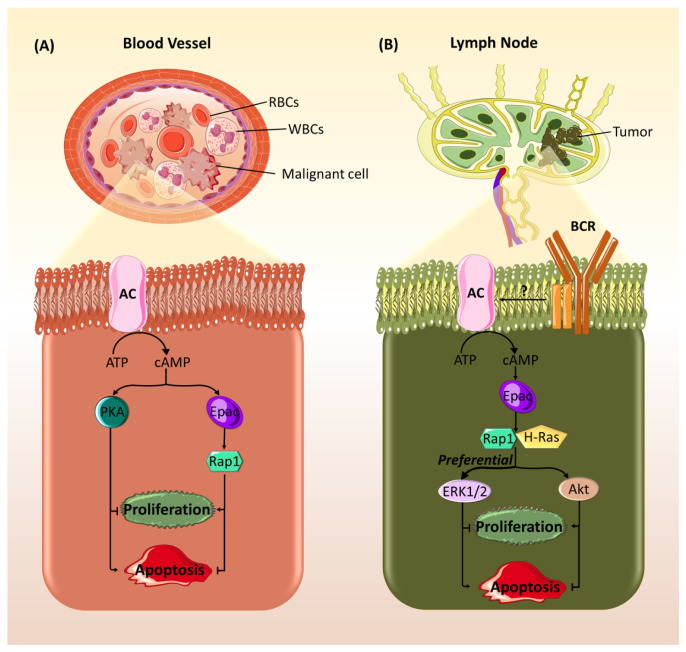
Epac has opposing effects on cell proliferation and apoptosis in different blood cancers types. (**A**) Epac enhances cell growth and survival and inhibits apoptosis in B cell chronic lymphocytic leukemia and acute lymphoblastic leukemia. cAMP can act through both downstream effectors, Epac and protein kinase A (PKA), that have contradictory effects. In contrast to PKA, Epac, through Rap1, plays an anti-apoptotic role and elicits pro-survival effects. (**B**) Epac promotes cell growth arrest and apoptosis in immature B cell lymphoma. Activation of B-cell antigen receptor (BCR) leads to activation of AC and a subsequent accumulation of cAMP. cAMP in its turn activates Epac, which acts through the small G proteins, Rap1 and H-Ras, to activate the pro-apoptotic ERK1/2 and the anti-apoptotic Akt. This activation seems to be more preferential towards ERK leading to a final result of growth arrest and increased apoptosis.

In addition to exhibiting opposing effects in different types of cancers, studies have shown that Epac could have contradictory effects among cell lines of the same cancer. For example, Epac, similar to PKA, has been found to mediate opposing effects on cell proliferation in different types of neuroendocrine tumors (NETs). In pancreatic-NET, Epac increases cell proliferation by increasing cyclin D1 levels and decreasing p27, a CDK inhibitor, levels, while it has opposite effects in bronchial carcinoids [57]. This cannot be explained by irrelevance of cyclin D, as its decrease has been reported in cancer cells of aggressive phenotypes [74], However, it could be explained by differences in the expression of Raf proteins. B-Raf is predominant in pancreatic-NET, while Raf1 is prominent in bronchial carcinoids. Previous studies have shown that cAMP activates MAPK, and thus cell proliferation, via signaling through B-Raf, whereas it inhibits MAPK through Raf1 [75,76]. Similarly, Epac has been found to promote opposing effects on the survival of blood cancers. In contrast to PKA, Epac promotes the survival of B-cell chronic lymphocytic leukemia (B-CLL) via the activation of Rap1 (Figure 4A) [49]. In addition, Epac opposes PKA’s pro-apoptotic role in acute lymphoblastic leukemia (ALL) cells and exerts a weak antagonistic effect in promoting cell survival (Figure 4A) [50]. On the other hand, in WEHI-231 immature B lymphoma cell lines, Epac mediates B-cell antigen receptor (BCR)-induced growth arrest and decreases cell survival by activating Rap1 and H-Ras and subsequently enhancing the activation of ERK1/2 and Akt (Figure 4B) [48].

## 3. Role of Epac in Cancer Cell Migration and Metastasis

Cell migration plays a crucial role in tumor invasion and metastasis. Similar to its role in cancer cell proliferation, the effects of Epac on cancer cell migration and metastasis is cell type specific. Studies have shown that Epac affects a diverse array of signaling pathways involved in tumor cell motility and invasion (Table 2). Some of the key pathways include cytoskeletal reorganization, extracellular matrix (ECM) and cell surface molecules, integrins activity/trafficking, and stress signals.

**Figure 5:**
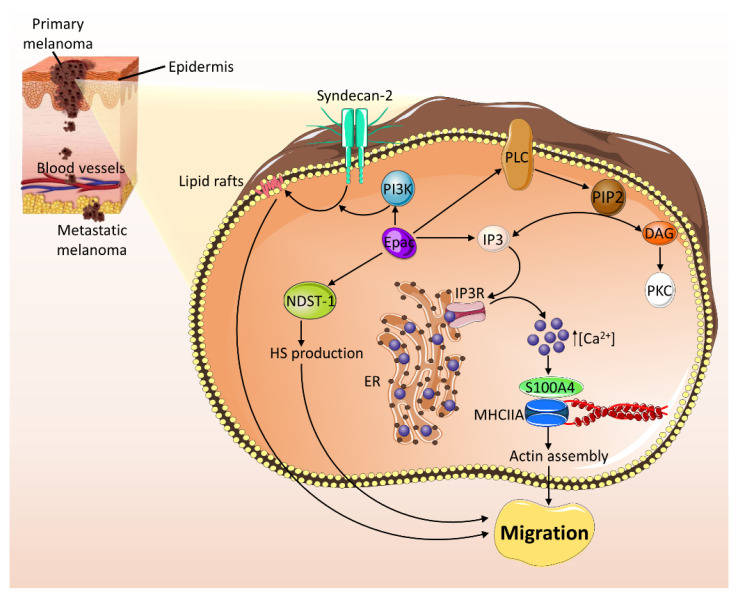
Epac promotes cell migration and metastasis in melanoma. Epac activates PI3K pathway, which promotes tubulin polymerization mediating the translocation of syndecan-2 into lipid rafts. Furthermore, Epac upregulates the expression of NDST-1, resulting in an increase in HS production. Both the translocation of syndecan-2 and the increase in HS production induce cell migration and metastasis. Epac also promotes melanoma cell migration and metastasis via a Ca^2+^-dependent mechanism. Epac activates phospholipase C (PLC), which catalyzes the breakdown of PIP2 into IP3 and DAG, activating the calcium signaling pathway. Epac can also activate inositol triphosphate (IP3). Activated IP3 bind to its receptor, IP3R1, on the membrane of ER, leading to the release of Ca^2+^. The increase in intracellular levels of Ca^2+^ enhances the interaction between S100A4 and MHCIIA, consequently promoting actin assembly and cell migration/metastasis. DAG: diacylglycerol; ER: endoplasmic reticulum; HS: heparan sulfate; IP3: inositol triphosphate; IP3R1: inositol 1,4,5-triphosphate receptor isoform 1; MHCIIA: myosin heavy chain IIA; NDST-1: N-deacetylase/N-sulfotransferase-1; PIP2: phosphatidylinositol 4,5-bisphosphate; PI3K: phosphoinositide 3-kinase; PKC: protein kinase C.

In melanoma, a plethora of studies confirm Epac’s pro-metastatic role (Figure 5). By activating Rap1, Epac induces the activation of ERK pathway and α_v_β_3_ integrin promoting tumorigenesis and migration in a number of melanoma cell lines [83]. Another study revealed a role of Epac in promoting melanoma migration and metastasis by enhancing the translocation of syndecan-2, a cell-surface heparan sulfate proteoglycan, to lipid rafts and increasing the production of heparan sulfate (HS), a major component of ECM [84]. Epac-mediated translocation of syndecan-2 is regulated by PI3K pathway activation, which promotes tubulin polymerization. Furthermore, Epac induces cell migration by upregulating the expression of N-deacetylase/N-sulfotransferase-1 (NDST-1), an enzyme that increases N-sulfation of HS and augmenting HS production [84,85]. In addition, Epac mediates endothelial cells angiogenesis and neighboring Epac-poor melanoma cells migration by cell–cell communication via fibroblast growth factor-2 (FGF-2)-HS interaction [86]. Besides the HS-related mechanism, a Ca^2+^-dependent role in Epac-mediated melanoma cell migration and metastasis has been reported [87]. In this regard, Epac activates phospholipase C/inositol triphosphate (PLC/IP3) receptor pathway, leading to intracellular Ca^2+^ release and elevation and enhanced interaction between S100A4, a Ca^2+^ binding protein, and the myosin heavy chain IIA isoform (MHCIIA). This interaction is known to promote actin assembly, which plays a role in cell migration [87,95,96]. Furthermore, a cross talk between Epac and G-protein βγ subunits (Gβγ) was later suggested in Ca^2+^ signaling and melanoma cell migration. By activating Ca^2+^ entry from the extracellular space, Gβγ inhibits Epac-induced cytosolic Ca^2+^ elevation and cell migration [88]. Interestingly, a recent study revealed an opposing role of Epac in melanoma progression, where Epac, via Rap1, plays a pro-survival role in primary melanoma and switches to an anti-survival role in metastatic melanoma [97]. The contradictory role of Epac in metastatic melanoma could be explained by the idea that proliferation is inhibited during metastasis to favor an invasive phenotype. Evidently, a state-switching model for melanoma progression was described by an in vitro study, which showed that changes in the tumor microenvironment and the gene expression switch the proliferative state into an invasive one [98].

**Figure 6:**
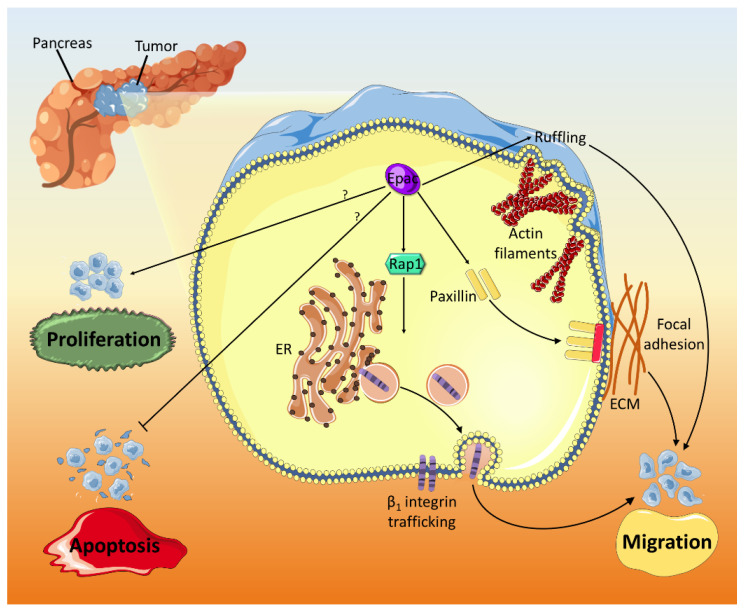
Epac has a positive role in pancreatic cancer progression. Epac increases cell ruffling and enhances the accumulation of paxillin near focal adhesion points to aid in their formation. Furthermore, Epac mediates β1 integrin trafficking in pancreatic malignant cells. All three mechanisms (ruffling, formation of new focal adhesions, and β1 integrin trafficking) stimulate cancer cell migration. Epac also promotes proliferation and attenuates apoptosis in pancreatic cancer through mechanisms that are yet to be identified.

Epac is also overexpressed in pancreatic cancer and mediates migration and invasion (Figure 6) [92,93]. The pro-migratory and invasive effects of Epac occur through Rap1 activation and integrin β1 trafficking facilitation, which is pivotal for cell migration [99,100]. Besides, Epac opposes PKA’s anti-migratory effects and promotes migration by modulating cell ruffling and paxillin accumulation in focal adhesions [94]. In non-small lung carcinoma (NSLC), Epac mediates PGE2- and isoproterenol (ISO)-induced cell migration (Figure 3B). PGE2 downregulates E-cadherin expression, upregulates β -catenin nuclear translocation and transcriptional activity, and induces cell migration [81]. PGE2-related effects are promoted through Gα_s_-coupled EP2 receptor and cAMP, which activates Epac, leading to its translocation to perinuclear regions [101,102]. In the nucleus, the association between Epac and β-catenin is mediated by Ezrin, a member of the AKAP family. Pharmacological and genetic knockdown of Epac confirmed its critical role in mediating PGE2-induced β -catenin activation and cell migration [81]. Epac, along with PKA, is also involved in ISO stress signaling-induced increase in histone deacetylase 6 (HDAC6) [82], a key regulator of cell migration [103]. By inhibiting c-Raf-MEK-ERK pathway, Epac/Rap1 induces an increase in HDAC6, which deacetylates α-tubulin, increases microtubules dynamics, and stimulates cell migration [82].

Additionally, Epac, in contrast to PKA, promotes cervical cancer migration via Rap1-mediated activation of Rac1 [79]. By enhancing the activity of Rac1, a small GTPase that is required for lamellipodia formation [104], Epac/Rap1 pathway might also contribute to increased invasiveness during tumor metastasis [79]. Similarly, Epac has been shown to promote cell invasion and metastasis in in vitro and in vivo fibrosarcoma models by activating Rac1 [80]. Epac/Rap1 and the subsequent activation of Rac1 mediate invadopodia formation by autotaxin (ATX) [80], an exoenzyme that increases aggressiveness and metastasis of many tumors [105,106,107], and lysophosphatidic acid (LPA4) receptor signaling [80]. The pro-migratory effects of Epac have been reported also in breast cancer cells. Indeed, pharmacological inhibition of Epac by treatment with the Epac specific inhibitor 09 (ESI-09) decreases the adhesion of cells to their substratum and disrupts microtubules distribution and AKAP9 localization, contributing to reduced breast cancer cell migration [78].

Although the majority of studies confirms a pro-migratory role of Epac in numerous carcinomas, a couple of studies illustrate an anti-migratory role. For example, Epac inhibits migration of bladder cancer cells. Overexpression of Epac in bladder tumor tissues specimens and cell lines activates Rap1, which probably alters cell morphology, enhances cell–cell adhesion, and impairs cell migration [77]. Similarly, Epac mediates norepinephrine-induced inhibition of migration in ES-2 ovarian carcinoma cells, a highly motile cell line, by activating Rap1 [89]. In a different ovarian carcinoma cell line, Ovcar3 cells, Epac/Rap1 has been shown to induce integrin-mediated cell adhesion to fibronectin [90] and laminin-5 [91]. Integrin-mediated cell adhesion has been shown to promote ovarian cancer cell invasion [108,109,110]. Taken together, it is reasonable to speculate that Epac could promote cancer cell migration and invasion in Ovcar3 cells through integrins. Nevertheless, the discrepancy in these observations may be attributed to a cell type-specific role of Epac.

## 4. Role of Epac in Cancer Cell Death

One of the important hallmarks that delineate malignancy is the decreased susceptibility to the different mechanisms of cell death, mainly apoptosis and autophagy [111]. Apoptosis is defined as the programmed suicide that a cell undergoes by terminating its growth and committing to a controlled death without spilling cellular content into its milieu [112]. Autophagy, on the other hand, is the process that gets rid of senescent cells by channeling cellular components into lysosomal degradation followed by recycling of the resulting molecules [112]. Failure of these two mechanisms in clearing stressed cells is an important step in malignant transformation [111]. Regulation of these mechanisms has been found to be highly affected by cAMP signaling. Both Epac and PKA have been proven to play myriad roles in controlling death of cancer cells. Contextually, Epac is able to transmit death signals in malignant cells or play a protective role and ensure their survival, depending on cancer cell type (Table 3).

The pro-apoptotic effects of Epac signaling can be seen in different types of cancer. Notably, Epac-mediated apoptosis appears to be tightly linked to downstream effectors, Rap1, ERK1/2, and Akt. Epac has been found to potentiate growth arrest and apoptosis initiated by BCR signaling in immature B cell lymphoma (WEHI-231 cell line) (Figure 4B) [48]. This pro-apoptotic function in immature B cell lymphoma is elicited through activation of Rap1 and H-Ras, which in their turn continue the signaling cascade by activating both ERK1/2 and Akt [48]. Given that ERK2 and Akt exhibit pro-apoptotic [114] and anti-apoptotic effects [115] in WEHI-231 cells, respectively, it seems likely that Rap1/H-Ras signaling skew the balance of Akt to ERK activation in favor of apoptosis. Furthermore, Epac elicits death-promoting signals in glioblastoma cells, where it acts synergistically with PKA in mediating rolipram-induced reduction of brain tumor size [51]. In contrast to Epac-mediated apoptosis in immature B cell lymphoma, Epac-promoted cell death in glioblastoma is probably driven by Rap1-induced inhibition of ERK [113], as ERK acts as an inhibitor of apoptosis in these cells [116]. Interestingly, Epac has been also shown to mediate autophagy and subsequent tumor size reduction in glial malignant cells of mice treated with tricyclic antidepressants [117].

On the other hand, Epac has been implicated in anti-apoptotic signaling in other types of cancer. In this sense, it has been shown that Epac mediates pro-inflammatory signals that are protective against apoptosis in prostate cancer. Epac anti-apoptotic effect occurs through ERK1/2 and Akt activation with subsequent mTOR induction (Figure 2) [61]. Additionally, Epac decreases apoptosis in breast cancer [78] and pancreatic cancer [59]. However, the exact signaling mechanisms are not well understood. Moreover, in B-CLL, Epac seems to promote anti-apoptotic effects, which are antagonized by PKA [49] (Figure 4A). Indeed, it is established that Epac has the capacity to mitigate the apoptotic effect of glucocorticoid treatment in ALL (Figure 4A). In a similar fashion to that observed in B-CLL, Epac anti-apoptotic role is opposed with that of PKA, whose expression is associated with sensitivity to glucocorticoids in ALL [50]. The discrepancy in Epac’s impact on apoptosis in the aforementioned studies may be attributed to differences among cell lines of the same cancer type.

Alternatively, Epac can also participate in indirect protection of cancer cells from death by means of immunomodulation. Regulatory-T cells (Tregs) are known to have a suppressive role against effector-T cells (Teffs). This aims to prohibit Teffs from executing their cytotoxic effects on the body’s own healthy cells [112]. Thus, increased activity of Tregs in the tumor microenvironment is associated with decreased ability of Teffs to kill malignant cells [118]. Epac regulates the suppressive ability of Tregs as well as the response of Teffs to this suppression [119]. This highly Epac-related attenuation of immune response manifests as a general protective role for cancer cells.

Obviously, most of the mentioned studies focused on apoptosis as the underlying cell death mechanism driven by Epac signaling. However, literature on autophagy in mediating Epac-provoked cell death in cancer is scarce. Based on previous observations [120], we hypothesize a positive correlation between Epac and autophagy in cancer cells. In this regard, pro-autophagic effects of Epac have been detected in hypertrophic cardiomyocytes [120]. The underlying signaling pathway has been found to involve Rap2B and PLC-mediated increase in intracellular Ca^2+^ concentration. Elevated Ca^2+^ levels ultimately lead to activation of AMP-dependent protein kinase (AMPK), which in turn inhibits mTOR1, subsequently mediating the autophagic events [120]. Interestingly, Epac/Rap2B/PLC-induced Ca^2+^ accumulation appears to elicit anti-autophagic signals in neurons with intracellular aggregated Huntingtin, where Ca^2+^ activates calpain, a known inhibitor of autophagy [121]. Notwithstanding, the Epac/Rap2B/PLC pathway and its context-dependent modulations of autophagy in cancer merit further investigation.

## 5. Epac and ROS: A Potential Interaction in Cancer

As previously established, Epac plays a pivotal role in the progression of several types of cancer by regulating an array of factors and pathways. Oxidative stress, through the production of reactive oxygen species (ROS), has been proposed as a key regulator of cancer development and progression [122]. Similar to Epac, ROS display paradoxical actions in cancer. Under normal physiological conditions, ROS homeostasis is maintained by the balance between ROS-producing enzymes and anti-oxidative ones [123]. However, in the context of cancer, elevated levels of ROS are prevalent in the malignant cells, where they promote the accumulation of genetic mutations, enhance survival and proliferation, and improve adaptation to hypoxia and metabolic stress [124]. Surprisingly, an excessive production of ROS does not magnify their pro-malignant capacity but rather produces anticancer effects [124]. This is attributed to the fact that uncontrolled increase of ROS can put cancer cells under high stress and induce apoptosis, autophagy, and necroptosis [122]. Although extensive efforts have been employed to explore the individual effects of Epac and ROS in cancer progression, very limited literature exists on the possible interplay between the two players to the best of our knowledge.

Conversely, an established interaction between Epac and ROS exists in cells and conditions other than cancer. It appears that Epac negatively regulates the production of ROS. For instance, under diabetic pathological conditions, Epac downregulates Src activation and its downstream pathway, PI3K/Akt, which in turn reduces ROS levels in pancreatic β-cells [125]. In addition, Epac decreases ROS levels and limits the degree of oxidative stress during ischemia-reperfusion injury [126]. The negative correlation between Epac and ROS may plausibly explain the mechanism underlying Epac maintenance of tubular epithelial cell adhesion during renal failure induced by ischemia-reperfusion injury [127]. In the heart, the Epac2–Rap1 axis decreases myocardial arrhythmia susceptibility by attenuating mitochondrial ROS production [128]. However, Epac-mediated inhibition of ROS production does not always result in favorable outcomes. For example, Epac downregulates ROS levels in neutrophils by inhibiting PLC and PKC activity. This allows the bacteria to escape the innate host defense mechanism of ROS-mediated killing [129].

Given their well-established roles in cancer cell proliferation, migration, and apoptosis, Epac and ROS seem likely to regulate each other. Indeed, in contrast to the CREB-mediated role of Epac [55,69], ROS reduces proliferation in lung cancer cells and promotes their death via apoptosis [130]. Since CREB is known to induce proliferation through its anti-oxidative role [131,132], this calls us to speculate that the role of Epac in lung cancer may be attributed to CREB-mediated inhibition of ROS production. Moreover, while Epac promotes migration and invasion in lung and breast cancers, ROS display contrasting actions [133,134]. Furthermore, Epac and ROS exert opposing effects on cell–cell adhesion and endothelial barrier in human umbilical vascular endothelial cells (HUVECs) [135,136,137]. Accordingly, it is tempting to speculate that, by inhibiting ROS-dependent decrease in cell–cell contacts and increasing in cell permeability, Epac would maintain cell–cell based adhesion and provoke migration/metastasis in certain cancer types. Taken together, these observations provide a new potential avenue of research to uncover the plausible negative interaction between Epac and ROS.

Interestingly, some studies have shown that Epac and ROS mediate similar effects. In prostate cancer, for example, Epac and ROS promote cell proliferation [64,138]. Epac elicits part of its effect by activating B-Raf, which appears to mediate similar effects in melanoma by increasing ROS production [139]. In addition, both Epac and ROS activate the ERK1/2 pathway and potentiate cancer cell migration and invasion [82,140,141]. Collectively, these observations reasonably imply that the differential effects of Epac on ROS may be cancer type-dependent and may even involve specific chemical reactive species production. Future research should focus on Epac and ROS interactions in cancer and identify the molecular mechanisms underlying their crosstalk.

## 6. Implication of Epac Modulators in Cancer Therapy

Epac has been implicated in a number of processes in malignant cells pertinent to proliferation, migration/metastasis, and apoptosis. Accordingly, Epac has been extensively explored as a potential target for cancer therapy. Indeed, several in vitro and in vivo studies demonstrate that Epac modulation can serve as a plausible novel modality in cancer treatment. Both Epac inhibitors [54,92] and activators [117] have displayed cancer type-dependent therapeutic value that is worth being more deeply investigated.

Besides being potential chemotherapeutic agents on their own, Epac modulators can be used as adjunctive drugs in cancer treatment plans. As previously mentioned, Epac exerts immunosuppressive effects in both Tregs and Teffs [119]. These effects not only affect the cytotoxic activity of host T cells to cancer cells but also interfere with the tumoricidal efficacy of T cell-based cancer immunotherapies [142]. This suggests that coupling immunotherapy with Epac inhibitors can spare injected T cells and Epac-mediated suppression and subsequently potentiate their action. In fact, the combination of Epac inhibitor, ESI-09, and lithium, a known anti-tumor treatment [143,144,145], results in a significantly greater inhibitory effect in comparison to each treatment alone [59]. This implies that Epac inhibitors could synergistically act with other chemotherapeutic drugs to treat cancer.

On the other hand, Epac activators can play a role in potentiating the effects of ionizing radiation and chemotherapeutic drugs, such as Topoisomerase II inhibitors, in tumor regression. It is well known that radiotherapy and certain chemotherapeutic agents have the ability to introduce lethal double-stranded DNA breaks in the genome of malignant cells. DNA-dependent protein kinase (DNA-PK) is involved in cellular attempts to fix these breaks and save themselves [146]. Studies have established that DNA-PK inhibitors can achieve chemo-sensitization and radio-sensitization of tumor cells both in vivo and in vitro [146]. Interestingly, Epac has been found to facilitate the nuclear exit of DNA-PK, separating the enzyme from its substrates and interfering with the correction of double-stranded breaks [147].

With the discovery of Epac as a central signaling effector, several molecules with Epac modulatory activity have been developed. Currently, several inhibitors and activators exist, and their medical significance is constantly being investigated. Epac inhibitors display specificity towards Epac over PKA, and some of them have well-established selectivity towards one of the two Epac isoforms [148]. These include ESI-09, which possesses a selective competitive antagonistic activity against Epac1. Contrarily, one study raised concerns that the effects elicited by ESI-09 are not selective and are due to its general protein denaturation abilities [149]. However, these concerns have been refuted by a subsequent investigation that established the selectivity of the drug and showed that its protein destabilizing effects are not significant at pharmacologically effective doses [150]. ESI-09 has been also proven to exhibit excellent bioavailability and a good safety profile when used in animal models [150]. Another Epac1 selective inhibitor is CE3F4R, which acts as an unconventional non-competitive inhibitor, which binds the Epac1-cAMP complex [151]. On the other hand, the molecules ESI-05 and ESI-07, which are Epac2 selective, have been shown to act on a recently identified allosteric site. This site falls on the interface between the two CNBDs that Epac2 possesses; meanwhile, it is absent in Epac1 due to the presence of one CNBD only. Binding of ESI-05 or ESI-07 to this domain locks Epac2 in its inactive state described previously [152].

In parallel, molecules with Epac activating capacity have been exploited. They also exhibit selective effects towards either of the two isoforms. A major group of these activators are cAMP analogues with modifications that render them unable to elicit similar activation in other cAMP-dependent proteins, mainly PKA. For instance, 8-pCPT-20-O-Me-cAMP has been proven to be a potent activator of Epac1 [148]. A more potent prodrug of this compound has been developed and is known as 8-pCPT-20-O-Me-cAMP-AM. This esterified prodrug lacks the negative charge present on 8-pCPT-20 -O-Me-cAMP and is readily hydrolyzed to the active form after crossing the cell membrane [148]. Besides Epac1 specific activators, Sp-8-Bnt-Me-cAMPS is a cAMP analogue that has been shown to achieve significant activation of Epac2 with poor activation of Epac1 [153]. Interestingly, a similar preferential activation has been demonstrated by sulfonylureas, which are drugs already approved for clinical use in diabetic patients [154,155].

The discussed drugs and their different interactions with Epac constitute a pool of promising potential targets in the treatment of cancer and in disabling the critical mechanisms that drive its survival and progression. What makes Epac modulators even more appealing is that they are expected to exhibit acceptable safety and limited side effects. This is based on the fact that each isoform of Epac shows differential distribution and can be targeted specifically and independently of the other [11,12]. Therefore, it is safe to speculate that inhibiting or activating one of them will confer effects that are specific to the targeted tissue-type with limited side effects. Moreover, Epac is highly expressed in cancer cells, and these cells become dependent on its effects. This has been shown not only in cell cultures [25] but also in descriptive studies on human patients. Indeed, a cohort study shows that levels of Epac were elevated in gastric cancer cells relative to other tissues in patients with the disease [54]. Similarly, overexpression of Epac was observed in breast tumor cells when compared to nonmalignant ones in the same patient [156]. Therefore, modulation of Epac activity will likely produce more potent effects in malignant cells than in normal ones. Finally, studies indicate that Epac-deficient mice do not show signs of failure to thrive. This suggests that Epac does not mediate important developmental functions and that modulating it will not produce deleterious effects [25]. Although the safety of drugs that act on Epac has not been well-investigated in a cancer setting, their side effects are reported to be minimal when used for cardiovascular diseases. In fact, studies suggest that the use Epac modulators poses a lower risk for heart failure than beta-blockers [157]. Likewise, these drugs have the potential to become alternative pain management medications to opioids due to their lesser complications [25]. Further research on in vitro and in vivo models is required to screen the potentials of these agents as chemotherapeutics. This will help pave their way into the final stage of clinical trials and ultimately to their integration in cancer treatment plans.

## 7. Concluding Remarks

The discovery of Epac has led to major advances in understanding the role of cAMP in cancer. Epac and PKA, the two main cAMP effectors, can act synergistically, antagonistically, or independently. It is now clear that Epac mediates many of the PKA-independent, cAMP-regulated functions. It is worth noting that the observations discussed in this review are attributed to the actions of Epac1. There is not enough evidence to support a role of Epac2 in cancer. However, one study has shown that Epac2 enhances cisplatin-induced apoptosis in lung cancer cells by promoting the accumulation of HDAC8. This accumulation is mediated by Rap1 activation, which in turn inhibits Akt and subsequently leads to halting JNK-controlled HDAC8 degradation [158]. Although the results of this study are intriguing, more studies are needed to shed light on the role of Epac2 in cancer. Additionally, considering that Epac1 and Epac2 isoforms exert, respectively, anti-apoptotic [55] and pro-apoptotic effects in lung cancer, it would also be of interest to study antagonistic or synergistic roles that the two isoforms may play in the other cancer types.

In conclusion, Epac has the ability to promote or inhibit cell proliferation, migration/metastasis, and apoptosis in a number of carcinomas. The contradictory effects of Epac in different cancer types and among different cell lines of the same cancer type demand focusing on downstream effectors that mediate Epac signaling to facilitate the development of new therapeutic strategies in cancer treatment. Unquestionably, future research, especially clinical research, is required to reproduce the results of in vitro and in vivo studies. 

## Figures and Tables

**Table 1:** Role of Epac in cancer cell proliferation and its downstream signaling targets.

Cancer Type	Proliferation	Signaling Target	References
**Blood cancers**			
Immature B cell lymphoma	Attenuates	Rap1/H-Ras/ERK/Akt	[48]
B-CLL	Promotes	Rap1	[49]
ALL	Promotes	*Unidentified*	[50]
**Brain cancer**	Attenuates	Rap1	[51]
		MAPK	[52]
**Breast cancer**	Promotes	*Unidentified*	[53]
**Gastric cancer**	Promotes	*Unidentified*	[54]
**Lung cancer**	Promotes	Rap1/Akt/CREB	[55]
	Promotes	XRCC1	[56]
**Neuroendocrine cancer**			
Pancreatic-NET	Promotes	Cyclin D1 and p27	[57]
Bronchial carcinoids	Attenuates	Cyclin D1 and P27	[57]
**Ovarian cancer**	Promotes	PI3K/Akt/Cyclin D1/CDK4	[58]
**Pancreatic cancer**	Promotes	*Unidentified*	[59]
**Prostate cancer**	Attenuates	MAPK	[60]
	Promotes	ERK/PI3K/mTOR	[61,62,63]
	Promotes	Cyclin B1 and CDK1	[64]
**Rectal cancer**	Promotes	Cyclin E1-Cnx43	[65]
**Renal cancer**	Attenuates	PI3K	[66]

**Table 2:** Role of Epac in cancer cell migration/metastasis and its downstream signaling targets.

Cancer Type	Migration/Metastasis	Signaling Target	References
**Bladder cancer**	Attenuates	Rap1	[77]
**Breast cancer**	Promotes	AKAP9	[78]
**Cervical cancer**	Promotes	Rac1	[79]
**Fibrosarcoma**	Promotes	Rac1/ATX/LPA_4_	[80]
**Lung cancer**	Promotes	β-catenin	[81]
		HDAC6	[82]
**Melanoma**	Promotes	α_v_β_3_ integrin	[83]
		Heparan Sulfate	[84,85,86]
		Ca^2+^	[87,88]
**Ovarian cancer**			
ES-2 cell line	Attenuates	Rap1	[89]
Ovcar3 cell line	Promotes	Integrins	[90,91]
**Pancreatic cancer**	Promotes	Integrin β1	[92,93]
		Cell ruffling/Paxillin/Focal adhesions	[94]

**Table 3:** Role of Epac in cancer cell apoptosis and its downstream signaling targets.

Cancer Type	Apoptosis	Signaling Target	References
**Blood cancers**			
ALL	Attenuates	*Unidentified*	[50]
B-CLL	Attenuates	Rap1	[49]
Immature B cell lymphoma	Promotes	Rap1/ERK/Akt	[48]
**Brain cancer**	Promotes	Rap1	[51,113]
**Breast cancer**	Attenuates	*Unidentified*	[78]
**Pancreatic cancer**	Attenuates	*Unidentified*	[59]
**Prostate cancer**	Attenuates	ERK/Akt/mTOR	[61]
